# Osteoporotic Burst Fracture in a Young Male Adult as First Presentation of a Rare PLS3 Mutation: A Case Report

**DOI:** 10.7759/cureus.51264

**Published:** 2023-12-29

**Authors:** Stefania Nikolaou, Ioannis Chatzikomninos, Ioannis Palavos, Paraskevi Langourani-Kosteletou, Kristallia Vitoula

**Affiliations:** 1 2nd Orthopaedic Department, KAT Attica General Hospital, Athens, GRC; 2 Spine and Scoliosis Department, KAT Attica General Hospital, Athens, GRC; 3 1st Orthopaedic Department, KAT Attica General Hospital, Athens, GRC; 4 Anesthesiology, Evangelismos General Hospital, Athens, GRC

**Keywords:** early-onset osteoporosis, osteoporotic spinal fracture, burst fracture, gene mutation, plastin-3, pls3

## Abstract

Low-impact spinal fractures in young patients are rare and should raise suspicion of an underlying condition, as these injuries are typically the result of high-energy trauma. We describe a case of a young male patient who sustained a burst fracture of the first lumbar vertebra (L1) following low-energy trauma. The patient underwent percutaneous posterior spinal instrumentation, yet the poor bone quality detected intraoperatively prompted further diagnostic evaluation. Subsequently, low bone mineral density (BMD) was detected, and a rare plastine-3 (*PLS3*) gene mutation was revealed in the genetic analysis. The patient was initiated on teriparatide therapy after the discovery of osteoporosis postoperatively. It is, therefore, imperative to investigate all young patients with low-energy spinal fractures preoperatively to discover the underlying pathology promptly.

## Introduction

Osteoporosis is a systemic skeletal disorder defined by low bone mass and microarchitectural deterioration of bone tissue, leading to bone fragility and an increased fracture risk. Early-onset osteoporosis (EOOP) is a term currently used to describe osteoporosis that develops in young adults and children [1]. Low-energy fractures occurring in childhood or early adulthood without any evident chronic illness require further investigation for an underlying genetic disease [2]. Recent evidence suggests that *PLS3 *mutations can cause early-onset osteoporosis. The gene codes for the protein plastin 3, an actin-binding and actin-bundling protein expressed in almost all human body solid tissues, which is believed to be involved in cytoskeleton remodeling. The gene’s X-chromosomal location explains why *PLS3* mutations affect mainly hemizygous males. In contrast, heterozygous females are less likely to present significant bone fragility [3]. We describe a case of a young male adult who experienced an osteoporotic spinal fracture as the first presentation of a rare *PLS3* mutation.

## Case presentation

**Figure 1:**
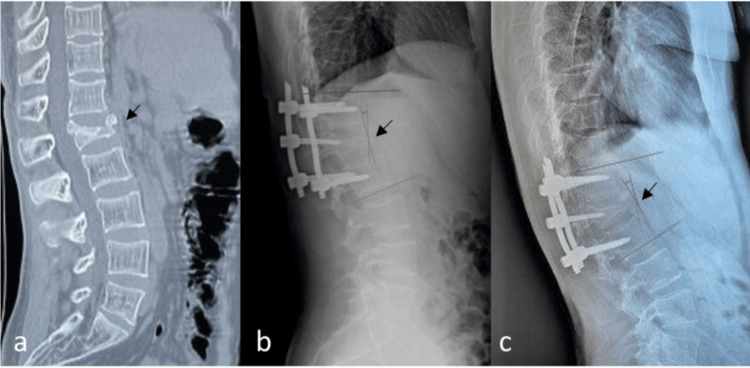
(a) CT scan of lumbar spine (sagittal plane), illustrating a burst fracture of L1 vertebra (black arrow). Loss of anterior vertebral body height: 70%, local kyphosis: 30°; (b) Immediate postoperative lateral plain radiograph indicating a residual kyphosis of 10°; (c) One-month follow-up lateral plain radiograph indicating a minor increase in local kyphosis of 5° (15° total).

A 26-year-old male was admitted to our department after sustaining a burst fracture of the L1 vertebral body (Figure 1a) during a physical altercation with his sibling (low-energy mechanism of injury). The precise circumstances of the injury occurrence were unclear by the information derived from the patient's history. The patient was neurologically intact and had no concomitant injuries. The medical record of the patient indicated diaphyseal fractures of both radius bones at the age of 13 and 15 years following sports injury. The patient's medical history involved no chronic disease or medication use.

The fracture was initially classified as an A3 incomplete burst fracture based on the AO Spine Thoracolumbar Injury Classification System and not as an osteoporotic fracture. Consequently, surgical treatment was decided due to severe local kyphosis (30° kyphosis at the thoracolumbar junction) and significant loss of vertebral body height (exceeding 50%) (Figure 1a). The patient was positioned in a prone hyperextended position under general anesthesia with pillows under the upper chest and pelvis to facilitate the postural reduction of the fractured vertebral body. Percutaneous posterior fixation was performed one level above and one level below the affected vertebra (T12-L2, short segment fixation). During the insertion of the pedicle screws, it was noted that the screws' grip was not as firm as typically expected for a patient of this age. Consequently, adequate fracture reduction by indirect techniques such as rod contouring could not be achieved intraoperatively. As a result, a residual kyphosis of 10° and a percentage of vertebral body height loss remained postoperatively (Figure 1b). In addition, a pullout of one screw was observed in intraoperative radiographs after the rod placement, compromising the stability of the fixation. The screw was replaced with a larger diameter one to ensure the stability of the construct. The poor bone quality identified intraoperatively, along with the mechanism of injury (low-energy fracture) in a young patient, raised the suspicion of an underlying condition and prompted further diagnostic evaluation

The patient underwent a full investigation, including laboratory tests (Table 1), serum immunofixation, and bone mineral density (BMD) test. The BMD result was 0.748 gr/cm2 (Z-score: -3.7) at the lumbar spine (LS), 1.665 gr/cm^2^ (Z-score: -2.9) at the femoral neck (FN), 0.678 gr/cm^2^ (Z-score: -3.0) at the total hip (TH) and 0.274 gr/cm^2^ (Z-score: -5.0) at the radius. BMD levels of the family members were measured, which revealed that the patient's brother had low BMD at LS, FN, and radius, and his father had osteopenia at LS and FN. However, the patient's sister and mother had normal BMD measurements. The results of the serum immunofixation test conducted on the patient revealed negative values for IgG, IgM, kappa, and lambda components.

**Table 1:** Laboratory test values

Laboratory parameter	Patient value	Normal range	Units
Hematocrit (Hct)	39.90	37-52	%
Hemoglobin (Hb)	13.40	12-18	g/dL
White Blood Cell Count (WBC)	9.22	4.6-10.2	x10^3/μL
Platelet Count (PLT)	272	130-400	X10^3/μL
Phosphorus (P)	4.2	2.5-4.7	mg/dL
Alkaline phosphatase (ALP)	66	40-150	IU/L
Triiodothyronine Free (FT3)	2.54	1.71-3.71	pg/mL
Thyroid-stimulating hormone (TSH)	2.13	0.35-4.94	μU/mL
Parathormone (PTH)	20.0	15.0-65.0	pg/mL
Vitamin D	31.8	Adequate: 30-60	ng/mL
Tryptase	4.1	˂ 11.4 μG/L	μG/L

Later, the patient was referred for an endocrinology assessment to investigate the underlying cause of the reported measurements. As part of the diagnostic assessment, the patient underwent a genetic analysis, which revealed a hemizygous mutation in the *PLS3* gene. The nucleotide change identified was c.827G>A, resulting in an amino acid change of p. Trp276Ter. According to the guidelines of the American College of Medical Genetics and Genomics/Association for Molecular Pathology (ACMG/AMP), this variant was a novel pathogenic mutation. Remarkably, this mutation has yet to be reported in the ClinVar and Decipher databases.

The patient had an uneventful postoperative course and was discharged on postoperative day 1. However, the radiological assessment performed during the one-month follow-up revealed partial loss of the initial reduction, indicated by a minor increase of local kyphosis at the fracture level (Figure 1c). However, the fixation remained stable after that, without any further progression of kyphosis, cutout, or pullout of pedicle screws, indicating successful surgical management. Eventually, despite percutaneous fixation, spinal fusion was achieved, and the patient returned to his pre-injury level of activities. Regardless of the residual kyphosis of 15°, which is poorly tolerated at the thoracolumbar junction, the patient had a good functional outcome one year postoperatively.

Additionally, the patient received teriparatide, calcium, and vitamin D as part of the treatment. Pharmacological therapy was initiated immediately after the diagnosis of *PLS3* gene mutation postoperatively. No new fractures occurred during a follow-up period of 18 months.

## Discussion

PLS3 is essential for the dynamic regulation of the actin cytoskeleton in the skeletal system, which can adjust to gravity force and mechanical stress modification through mechanotransduction. Mechanotransduction is the translation of mechanical signals into cellular responses, and its dysregulation is strongly linked with bone-related diseases. *PLS3* variants may cause alteration of PLS3 function and expression, leading to osteoporosis. Abnormalities in *PLS3* can disrupt the balance in bone homeostasis, which may result in the development of bone disease [4]. In 2013, Dijk et al. were the first to report a new monogenic form of osteoporosis caused by a *PLS3* gene mutation that mainly affected boys and men in five families [2]. Since then, several other families and individuals have been identified. It has become clear that *PLS3* mutations in affected males lead to severe, early-onset progressing osteoporosis, characterized by multiple vertebral compression fractures [1,2].

In the presented case, the patient's young age, combined with the absence of a chronic illness, medication use, and the unclear circumstances of the injury mechanism, did not raise suspicion at first of an underlying pathology. Consequently, the fracture was managed as an unstable A3 incomplete burst fracture based on the AO Spine Thoracolumbar injury Classification System and not as an osteoporotic burst fracture. Therefore, the patient underwent short fusion percutaneous fixation with solid spinal pedicle screws. Indirect reduction was performed intraoperatively by attempting to correct the kyphotic angle and the vertebral body height. However, the poor purchase of screws in bone took us by surprise. The poor bone quality detected intraoperatively raised suspicion for further investigation postoperatively. As a result, adequate fracture reduction could not be achieved intraoperatively. Luckily, our patient had a good functional outcome and returned to his pre-injury level of activities. Nonetheless, this may not have been the case. Fixation failure requiring revision surgery might have occurred if the progression of kyphosis persisted since the fracture was not appropriately managed as an osteoporotic fracture.

It is widely recognized that surgical treatment of osteoporotic fractures in the spine is challenging due to limited bone stock. The reduced bone density offers poor purchase for the instrumentation, increasing the risk of fixation failure. Implant failure in osteoporotic bone is often presented by pedicle screws loosening and backing out. Non-union is another common complication of fragility fractures [5]. Due to the reasons above, surgical management of osteoporotic spinal fractures differs significantly from traumatic spinal fractures.

First, multiple-level fixation is recommended to improve stability during instrumentation (long-segment fixation). In this way, stress is distributed evenly along the construct [6].

In addition, utilization of fenestrated pedicle screws is advised instead of solid ones. Polymethylmethacrylate augmentation (PPMA) procedures can be performed using fenestrated pedicle screws designed for cement injection [7]. Biomechanical studies have shown that fenestrated screws are more resistant to pull-out when compared to solid ones [5]. In osteoporotic bone, a gap is created between the threaded portion of the screw and the trabecular bone. Cement strengthens the bone/metal interface in such a gap. In a study conducted by Amendola et al., a group of patients with limited bone stock due to osteoporosis or tumor underwent posterior stabilization with fenestrated pedicle screws and PMMA augmentation. During the follow-up, it was concluded that there was a significant improvement in pain and function, and no incidents of screw pull-out were noted [8]. To optimize screw purchase, utilization of hydroxyapatite-coated screws, expandable screws, and larger diameter screws that increase the pull-out strength has been proposed [5].

Kyphoplasty is also an effective procedure to reduce fracture deformity by restoring vertebral body height in osteoporotic fractures. A cadaveric study by Hiwatashi et al. found that vertebral body height was restored up to 93% of the original vertebral body height after kyphoplasty. The wedge angle also decreased, improving coronal alignment [9]. By restoring vertebral height, sagittal balance is also improved in cases of kyphotic deformity [10].

A different surgical technique would have been chosen if the fracture had been classified as osteoporotic before surgery. Appropriate surgical treatment would have resulted in better fracture reduction, reduced surgical time, and significantly minimized the risk of postoperative complications. Furthermore, the unexpected intraoperative challenge caused by the poor bone quality identified during the surgical procedure would have been avoided by meticulous patient exploration preoperatively.

Percutaneous fixation is the optimal surgical treatment in cases of burst osteoporotic fractures [11]. However, long-segment fixation (two levels above and two levels below the fracture level) would have been a preferred option. Additionally, utilizing fenestrated screws instead of solid ones would have enhanced the stability of the construct. Another option would have been short-segment fixation combined with kyphoplasty, which theoretically combines the advantages of kyphoplasty by restoring the vertebral body height, and short-segment fusion, which is a relatively less invasive procedure [6].

Through this report, we aim to raise awareness in spine surgeons of the potential of an underlying rare genetic mutation in *PLS3* when a young patient presents with a low-energy spinal fracture. Osteoporotic vertebral fractures are infrequent in young adults [12]. Specifically, burst fractures in this age group are typically the result of high-energy trauma such as a fall from height or motor vehicle accidents [13]. Thus, there is a high probability to misdiagnose a burst fracture in a young patient without a history of chronic illness as traumatic instead of osteoporotic. Prompt identification of osteoporosis by BMD measurements using dual X-ray absorptiometry (DEXA) [14] is crucial to avoid unexpected sequelae. Additionally, the possibility of adverse postoperative complications, including fracture nonunion, fixation failure, or revision surgery, can be minimized. Referral for endocrine assessment and further diagnostic evaluation, including genetic analysis and initiation of proper pharmacological treatment, is essential for appropriate patient management.

## Conclusions

Young patients who present with low-energy spinal fractures should raise suspicion of an underlying pathology. Prompt identification of osteoporosis preoperatively is imperative for proper fracture classification and for the patient to receive the optimal surgical treatment. Further diagnostic evaluation and genetic analysis to uncover the underlying cause of osteoporosis is also necessary. In the presented case, the patient revealed a rare *PLS3* gene mutation after the medical investigation and received the optimal treatment, according to the endocrine assessment.
