# Correction: Osteoporotic Burst Fracture in a Young Male Adult as First Presentation of a Rare PLS3 Mutation: A Case Report

**DOI:** 10.7759/cureus.c152

**Published:** 2024-01-17

**Authors:** Stefania Nikolaou, Ioannis Chatzikomninos, Ioannis Palavos, Paraskevi Langourani-Kosteletou, Kristallia Vitoula

**Affiliations:** 1 2nd Orthopaedic Department, KAT Attica General Hospital, Athens, GRC; 2 Spine and Scoliosis Department, KAT Attica General Hospital, Athens, GRC; 3 1st Orthopaedic Department, KAT Attica General Hospital, Athens, GRC; 4 Anesthesiology, Evangelismos General Hospital, Athens, GRC

This article has been corrected at the request of the authors to remove the third author as well as Figures 2 and 3. The third author did not approve of the submission or publication of the article and had not provided the authors with permission to publish the results of the genetic analysis.

